# Dietary Energy Modulation and Autophagy: Exploiting Metabolic Vulnerabilities to Starve Cancer

**DOI:** 10.3389/fcell.2020.590192

**Published:** 2020-11-05

**Authors:** Alyssa J. Cozzo, Michael F. Coleman, Jane B. Pearce, Alexander J. Pfeil, Suhas K. Etigunta, Stephen D. Hursting

**Affiliations:** ^1^Department of Nutrition, The University of North Carolina at Chapel Hill, Chapel Hill, NC, United States; ^2^Duke University School of Medicine, Durham, NC, United States; ^3^Lineberger Comprehensive Cancer Center, The University of North Carolina at Chapel Hill, Chapel Hill, NC, United States; ^4^Nutrition Research Institute, The University of North Carolina at Chapel Hill, Kannapolis, NC, United States

**Keywords:** cancer, autophagy, fasting, metabolism, caloric restriction, cancer therapy

## Abstract

Cancer cells experience unique and dynamic shifts in their metabolic function in order to survive, proliferate, and evade growth inhibition in the resource-scarce tumor microenvironment. Therefore, identification of pharmacological agents with potential to reprogram cancer cell metabolism may improve clinical outcomes in cancer therapy. Cancer cells also often exhibit an increased dependence on the process known as autophagy, both for baseline survival and as a response to stressors such as chemotherapy or a decline in nutrient availability. There is evidence to suggest that this increased dependence on autophagy in cancer cells may be exploitable clinically by combining autophagy modulators with existing chemotherapies. In light of the increased metabolic rate in cancer cells, interest is growing in approaches aimed at “starving” cancer through dietary and pharmacologic interventions that reduce availability of nutrients and pro-growth hormonal signals known to promote cancer progression. Several dietary approaches, including chronic calorie restriction and multiple forms of fasting, have been investigated for their potential anti-cancer benefits, yielding promising results in animal models. Induction of autophagy in response to dietary energy restriction may underlie some of the observed benefit. However, while interventions based on dietary energy restriction have demonstrated safety in clinical trials, uncertainty remains regarding translation to humans as well as feasibility of achieving compliance due to the potential discomfort and weight loss that accompanies dietary restriction. Further induction of autophagy through dietary or pharmacologic metabolic reprogramming interventions may enhance the efficacy of autophagy inhibition in the context of adjuvant or neo-adjuvant chemotherapy. Nonetheless, it remains unclear whether therapeutic agents aimed at autophagy induction, autophagy inhibition, or both are a viable therapeutic strategy for improving cancer outcomes. This review discusses the literature available for the therapeutic potential of these approaches.

## Introduction

The overarching term *autophagy* is generally recognized to encompass three distinct processes: macroautophagy, microautophagy, and chaperone-mediated autophagy. Macroautophagy utilizes an isolation membrane called an *autophagosome* to sequester and transport protein aggregates or organelles to lysosomes for degradation (Mizushima et al., 2004; Kuma and Mizushima, 2010). In contrast, microautophagy involves direct engulfment of cytoplasmic components through invagination of the lysosomal membrane, while chaperone-mediated autophagy targets select cytosolic proteins and translocates them to the lysosome in a chaperone-dependent manner (Mizushima and Komatsu, 2011; Shaid et al., 2013). In this review, we will focus on pharmacologic and dietary approaches that have been examined for their potential to modulate dependence of cancer cells macroautophagy, referred to hereafter simply as autophagy.

In a growing tumor, cancer cells are faced with increased metabolic demands in a microenvironment characterized by dysfunctional vascularization, hypoxia, and fierce competition for a limited supply of nutrients (Dewhirst et al., 2008; Zhang et al., 2008). Under the harsh conditions of the tumor microenvironment, the highly conserved catabolic process of autophagy can support cancer cell metabolism through supply of critical metabolites via degradation and recycling of precise cargo such as misfolded proteins, dysfunctional mitochondria, and pathogens, as well as non-selective engulfment of bulk cytoplasmic components (Nakatogawa et al., 2009; Kuma and Mizushima, 2010; Boya et al., 2013; Shaid et al., 2013). Early studies investigating the effects of autophagy inhibition have utilized genetic silencing of key autophagy genes, effectively disrupting the autophagy cascade and providing more insight into the roles of autophagy in cancer initiation and aggressive features in cancer cells (Cufi et al., 2012).

Dietary interventions that restrict caloric intake may induce autophagy in normal and/or cancerous cells, and there is increasing interest in using these interventions clinically with the ultimate goal of manipulating systemic fuel availability to “starve” a developing tumor. Herein we discuss the roles of autophagy in cancer initiation, tumor progression, and therapeutic response. In addition, we provide: (i) an overview of the underlying molecular biology following restriction of dietary energy intake through approaches such as caloric restriction and various forms of fasting; (ii) we summarize the limited evidence from associated clinical trials that have utilized these interventions as an approach to improving treatment outcomes or reducing the toxic side effects of chemotherapy (Raffaghello et al., 2008; Lee et al., 2010); (iii) we address some of the currently available pharmacological approaches for both induction and inhibition of autophagy; and (iv) we briefly discuss the potential for synergy between dietary or pharmacologic energy manipulation and autophagy inhibition.

## Metabolic and Hormonal Regulation of Autophagy

Basal autophagy occurs constitutively through the signaling of hormones and growth factors (Rabinowitz and White, 2010), facilitating the maintenance of cellular homeostasis by removing redundant or damaged organelles and generating metabolites used to provide energy to the cell or create new macromolecules (Boya et al., 2013). In contrast, autophagy is induced above basal levels under conditions associated with cellular stress or low energy status, including a high AMP/ATP ratio, nutrient deprivation, and/or reduced growth factor signaling (Saha et al., 2018). The principal cellular regulators of autophagic flux are AMP-activated protein kinase (AMPK) and mechanistic target of rapamycin (mTOR), both of which function to integrate nutrient and energy signaling with cellular metabolism and various forms of fasting (Meijer and Codogno, 2011; Mihaylova and Shaw, 2011; Kim and Guan, 2015).

AMPK is an evolutionarily conserved serine/threonine protein kinase that acts as a key sensor of cellular energy status. Upon activation, AMPK works to restore energy homeostasis by activating an array of catabolic pathways including autophagy, as well as phosphorylating and inactivating mTOR (Hardie et al., 2016). High AMP/ATP ratios and glucose deprivation are the primary signals for AMPK activation (Gowans et al., 2013; Zhang et al., 2017).

**FIGURE 1:**
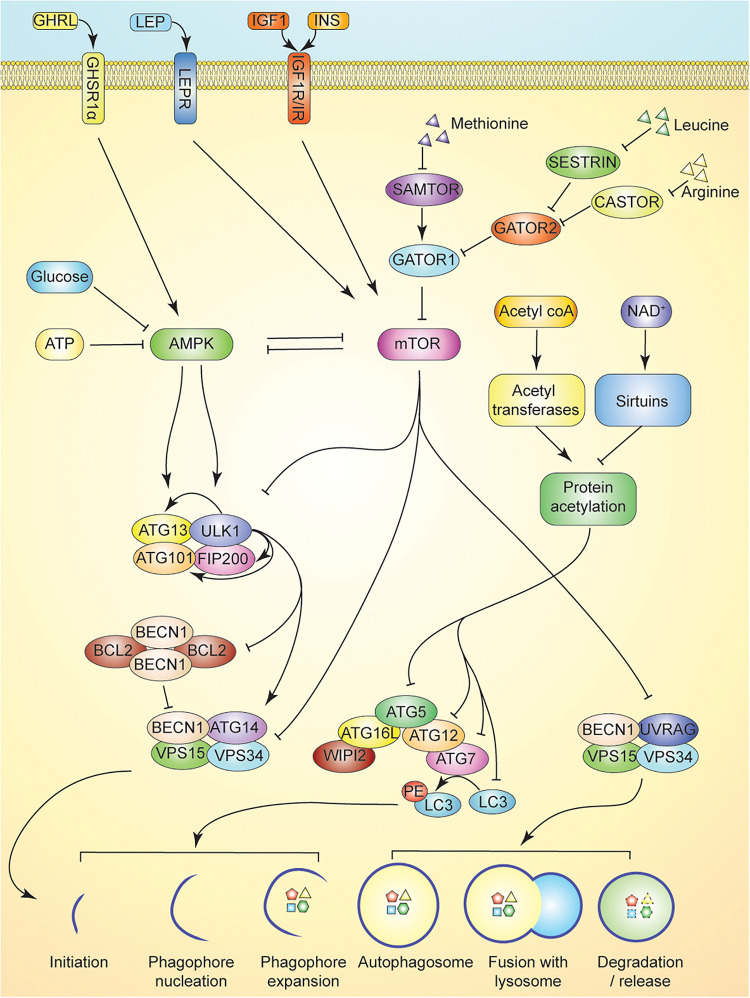
Overview of autophagy and its regulation. Nutrient sensing in autophagy induction is multifaceted. Activation of the ULK serine threonine kinase complex induces autophagy by promoting release of BECN1 from BCL2 inhibitory heterotetramers, and promoting the association of BECN1 with ATG14, VSP15, and VSP34 in Class III PI3K complex I. This complex is responsible for initiating isolation membrane formation. AMPK activation in response to cellular energy status activates ULK complex by phosphorylation of ULK1 and ATG13. Activation of autophagy is antagonized by mTOR inhibition of AMPK, ULK complex, and Class III PI3K complex I and II. mTOR activity is maintained by intracellular leucine, arginine, and methionine levels. Leucine and arginine inhibit SESTRIN and CASTOR, respectively, to promote GATOR2 inhibition of GATOR1, a key negative regulator of mTOR activity. Methionine, through production of SAM inhibits SAMTOR to suppress GATOR1 activity. Activation of growth factor signaling via hormones upstream of mTOR (e.g., leptin, insulin, and IGF1) further suppresses autophagy. GHRL signaling in contrast can activate AMPK to promote autophagy. Maintenance of protein acetylation by acetyltransferases is enabled by ready supply of acetyl-coA and suppresses the activity of ATG5-ATG12 complex further limiting autophagy induction. Activation of sirtuins by elevation of NAD^+^ levels promotes autophagy by reducing such inhibitory acetylation and enabling ATG5-ATG12 complex to lipidate LC3.

mTOR, also a serine/threonine kinase, is a master regulator of cellular growth and proliferation in response to nutrient and hormone signaling; namely, amino acid concentrations and insulin-like growth factor 1 (IGF1) and/or insulin levels (Avruch et al., 2006). In order to activate downstream anabolic pathways, mTOR complex 1 (mTORC1) must be recruited to the lysosome (Lawrence and Zoncu, 2019). The protein complex GATOR1 functions to inhibit mTOR activation via GTP hydrolysis of the heterodimeric Rag GTPases responsible for recruiting mTOR to the lysosomal surface (see Figure 1; Bar-Peled et al., 2013; Panchaud et al., 2013). The activity of GATOR1 is regulated by amino acid concentrations—specifically levels of leucine, arginine, and methionine. Leucine and arginine, functioning through SESTRIN and CASTOR respectively, interact with GATOR2 to inhibit mTOR upon amino acid deprivation (Kim J. S. et al., 2015; Saxton et al., 2016; Wolfson et al., 2016). Leucyl-tRNA synthetase (LRS) functions as another leucine sensor and positive regulator for mTORC1 as a GTPase-activating protein (GAP) for RagD (Lee et al., 2018). SAMTOR, an inhibitor of mTOR and sensor for S-adenosylmethionine, is responsible for mTOR inactivation in the context of methionine deprivation (Figure 1), which improves insulin sensitivity and extend lifespan in rodents (Orentreich et al., 1993; Gu et al., 2017).

Growth factor signaling involving insulin and IGF1 is another well-established upstream regulator of mTOR that further integrates host nutrient status with cellular metabolism. Both insulin and IGF1 activate the PI3K/AKT signaling axis upon binding to their tyrosine kinase receptor, resulting in increased activation of mTORC1 at the lysosomal surface (Saxton and Sabatini, 2017). Insulin, a peptide hormone produced by pancreatic β-cells, is released in response to elevated blood glucose (Braun et al., 2011). Hyperglycemia is a hallmark of metabolic syndrome and is associated with insulin resistance, aberrant glucose metabolism, chronic inflammation, and the production of other metabolic hormones such as IGF1, leptin, and adiponectin (Braun et al., 2011). IGF1, a peptide growth factor produced primarily by the liver, is typically bound to IGF binding proteins (IGFBPs), which regulate the amount of free IGF1 bioavailable to bind to the IGF1 receptor (IGF1R) to induce growth or survival signaling (Pollak, 2012). In metabolic syndrome, the amount of bioavailable IGF1 increases via hyperglycemia-induced suppression of IGFBP synthesis and/or hyperinsulinemia-induced promotion of hepatic GH receptor expression and IGF1 synthesis (Braun et al., 2011). Elevated circulating IGF1 is an established risk factor for many cancer types (Pollak, 2012).

Autophagy is initiated by the activation of Unc-51 like autophagy activating kinase 1 (ULK1). Upon activation, ULK1 phosphorylates autophagy-related protein 13 (ATG13) and focal adhesion kinase family interacting protein of 200 kD (FIP200), promoting the association of a protein complex involving ULK1 and the non-catalytic subunits ATG13, FIP200, and ATG101 (Hurley and Young, 2017). This ULK1 signaling complex links cellular energy status with autophagy induction, as AMPK activates the complex by binding and phosphorylating ULK1 on S317 and S777, while mTOR phosphorylates S757, blocking ULK1 association with AMPK (Kim et al., 2011). Thus, ULK1 signaling is responsive to both ATP levels (through AMPK) and amino acid levels (through mTOR) (Mihaylova and Shaw, 2011; Meijer et al., 2015).

Activation of the ULK1 complex initiates the formation of the phagophore, which requires translocation of the complex to an endoplasmic reticulum domain enriched for the lipid phosphatidylinositol 3-phosphate [PI(3)P] (Axe et al., 2008; Itakura and Mizushima, 2010). ULK1 also promotes the activation of Beclin 1 (BECN1)-containing PI3K class III complexes by disrupting the formation of inhibitory BECN1/BCL2 heterotetramers (Pattingre et al., 2005). Two distinct PI3K complexes, consisting of BECN1, vacuolar protein sorting protein 15 (VPS15), VPS34 and either ATG14 (complex I) or UV radiation resistance-associated gene protein (UVRAG) (complex II), are critical to phagophore initiation and autophagosome maturation, respectively (Backer, 2016). BECN1 and ATG14 on the PI3K class III complex I are phosphorylated by ULK1, activating the complex. Activation and recruitment of PI3K complex 1 to the site of autophagosome formation drives nucleation of the phagophore membrane and generation of PI(3)P, which is essential for recruiting additional ATG proteins and PI(3)P effectors, such as WIPI (Lamb et al., 2013). PI3K class III complex II promotes downstream fusion of the autophagosome with an endosome-lysosome, resulting in the breakdown of sequestered cellular components (Liang et al., 2008). mTOR directly inhibits the lipid kinase activity of both PI3K class III complexes through phosphorylation of ATG14 and UVRAG (Yuan et al., 2013; Kim Y.M. et al., 2015).

Following nucleation of the phagophore via the PI3K class III complex I, two conjugation systems involving ubiquitin-like proteins associate with the membrane to aid in phagophore expansion and autophagosome formation. ATG12 is first activated by ATG7 before binding irreversibly to ATG5, which interacts further with a small coiled-coil protein, ATG16, to form the larger ATG12-ATG5-ATG16L complex (Mizushima et al., 2011). The complex is recruited to the phagophore membrane and functions as an E3-like ligase to mediate the lipidation of microtubule-associated protein light chain 3 (LC3) with phosphatidylethanolamine (PE) (Fujita et al., 2008). LC3-PE can be localized to both the inner and outer membranes of the autophagosome, and upon autophagosome maturation, the lipidated LC3 on the outer membrane gets deconjugated by Atg4 (Chen and Klionsky, 2011). The ATG proteins then dissociate from the membrane before its closure into an autophagosome, while lipidated LC3 remains attached to the inner surface of the autophagosome. LC3 is believed to aid in expansion and closure of the isolation membrane, and is a widely used marker for identifying autophagosomes and monitoring autophagic flux (Tanida et al., 2004). LC3 also serves as a binding motif for multiple mitophagy-associated receptors such as BNIP3 and FUNDC1, allowing for delivery of the autophagosome membrane to the mitochondria for receptor-mediated mitophagy (Liu et al., 2012; Wu et al., 2014). LC3 also plays a critical role in ubiquitin mediated autophagy/mitophagy, where it binds LC3 interacting region (LIR) motifs of proteins such as p62 (SQSTM1, Sequestosome 1), OPTN (Optineurin), and NBR1(NBR1 Autophagy Cargo Receptor) which serve as a bridge between ubiquitinated cargo and autophagy machinery (Chen et al., 2019).

## Autophagy and Cancer

### Autophagy in Cancer Initiation

Basal autophagy exerts a protective role in suppressing malignant transformation and early tumorigenesis by regulating cellular homeostasis and metabolism through the degradation of intracellular components (Yun and Lee, 2018). Autophagy was initially thought to be a tumor suppressive mechanism because BECN1, key in phagophore formation, is a haploinsufficient tumor suppressor with monoallelic loss in several human breast, prostate, and ovarian cancers (Liang et al., 1999; Qu et al., 2003). However, this finding is confounded by the location of *BECN1* adjacent to the well established tumor suppressor breast cancer 1, early onset (*BRCA1*) on chromosome 17q21. Nonetheless, the cellular “quality control” resulting from the unfolded protein response, preservation of genomic stability, and prevention of reactive oxygen species (ROS) accumulation point to autophagy as a mechanism suppressing cancer initiation (Bhutia et al., 2013; Yun and Lee, 2018). Cancer cells exhibit reduced proteolysis or autophagic activity when compared with non-transformed cells (Gunn et al., 1977; Kisen et al., 1993; Bhutia et al., 2013).

Although human cancers largely lack evidence of genetic inactivation of core autophagy machinery, various murine models have revealed that knockouts of key autophagic genes promote tumorigenesis (Amaravadi et al., 2016). In addition to its role in initiating autophagy (Vega-Rubín-de-Celis, 2019), BECN1 is essential for early embryonic development and regulates growth factor receptor signaling (Yue et al., 2003; Rohatgi and Shaw, 2016). As a result, biallelic deletions of BECN1 cannot be studied because of lethality in animal models. In a model of immortalized mouse mammary epithelial cells (IMMECs) in nude mice, monoallelic BECN1 loss increased sensitization to metabolic stress, induced DNA damage response, and stimulated gene amplification in support of mammary tumorigenesis (Karantza-Wadsworth et al., 2007). Furthermore, *BECN1* overexpression in MCF7 breast carcinoma cells reduced tumorigenesis in nude mice (Liang et al., 1999). Moreover, *BECN1* heterozygosity in MMTV-*Wnt1* mice revealed increased WNT-1 driven mammary tumorigenesis compared with wildtype controls (Cicchini et al., 2014). Similarly, mice with either a monoallelic deletion for autophagy and Beclin1 regulator 1 (*Ambra1*) or a biallelic deletion for SH3 Domain Containing GRB2 Like, Endophilin B1 (*SH3GLB1*, aka *Bif-1*) revealed higher rates of spontaneous tumor incidence (Cianfanelli et al., 2015). UVRAG, another critical autophagy protein, is a component of Class III PI3K complex II, and activates BECN1 to enable phagophore formation (Liang et al., 2006). Mutated UVRAG has been reported to suppress autophagy and promote tumor growth in colorectal cancers (He et al., 2015).

The role of autophagy in early breast tumorigenesis remains unresolved. Murine models of hereditary breast cancer showed that monoallelic loss of *BECN1* reduces tumorigenesis and facilitates p53 induction (Huo et al., 2013). Similarly, *Palb2*^*f/f*^; *Wap*-*cre* mice with monoallelic loss of *BECN1 (BECN1^±^*) experienced a significant delay in mammary tumor formation compared with mice homozygous in *BECN1* expression (Huo et al., 2013). Additionally, Gong et al. (2013) showed that BECN1 is essential for the tumorigenicity of breast cancer stem-like cells. BECN1 competes with myeloid cell leukemia sequence 1 (MCL1), an antiapoptotic BCL2 family member, for stabilization by a common deubiquitinase, USP9X (Elgendy et al., 2014). Accordingly, loss of BECN1 may result in MCL1 accumulation (Elgendy et al., 2014). Hence, BECN1 may regulate breast cancer initiation through an autophagy-independent pathway.

Genetic disruption of other autophagy-related genes has also revealed autophagy-associated regulation of cancer initiation in other cancer types. ATG7 is essential for hematopoietic stem cell (HSC) maintenance, and deleting ATG7 in LSK (Lin^–^Sca-1^+^c-Kit^+^) cells resulted in HSC dysfunction, increased DNA damage, elevated reactive oxygen species (ROS), and myeloproliferation. Histologically, the infiltrating myeloid cells in these ATG7-deficient mice reportedly bear semblance to acute myeloid leukemia (Mortensen et al., 2011). Additionally, mice with systemic mosaic deletion of *Atg5* and liver specific *Atg7* deletion develop benign liver tumors more frequently than wildtype control mice (Takamura et al., 2011). Impairing autophagy through *in vivo* tissue-specific deletion of *Atg7* in pancreatic epithelial cells revealed increased inflammation, ROS accumulation, and mitochondrial damage, markers of oxidative stress that are well known risk factors for promoting cancer initiation (Antonucci et al., 2015).

Autophagy may also protect against cancer via suppression of oxidative stress via modulation of nuclear factor erythroid 2-related factor 2 (Nfe2l2/Nrf2)/kelch-like ECH-associated protein 1 (Keap1) and SQSTM1/p62 pathway (Komatsu et al., 2010; Jiang et al., 2015). p62 is a selective substrate of autophagy and cargo adapter that can disturb the Nfe2l2-Keap1 association, leading to the selective degradation of Keap1 and translocation of Nfe2l2 to activate antioxidant stress response genes (Taguchi et al., 2012). Under normal conditions, p62 is degraded by autophagy via its LC3 interaction region (LIR), but impaired autophagy leads to the accumulation of oncogenic p62 aggregates (Pankiv et al., 2007). Thus, the connection of oxidative stress to cancer promotion and the abnormal accumulation of p62 in several breast (Thompson et al., 2003; Li S. S. et al., 2017) and other cancers (Kitamura et al., 2006; Inoue et al., 2012; Saito et al., 2016) may in part explain the tumor suppressive effects of autophagy.

### Autophagy Dependence in Cancer Malignancy and Response to Therapy

In contrast to the protective role of autophagy in maintaining function and integrity in normal cells, following transformation, autophagy promotes progression and metastasis in several cancer types, thus revealing the “double edged” role of autophagy in cancer (Huo et al., 2013). In established tumors, autophagy may also act as an essential adaptive response to promote growth and overcome cellular stressors (White, 2015). For example, a variety of human cancers with mutations in the oncogene *Ras*—including pancreatic ductal adenocarcinoma (PDAC), bladder, large cell lung, colon, and prostate cancers—have high levels of basal autophagy *in vivo* even under nutrient-replete conditions, and are subsequently more sensitive to pharmacological autophagy inhibition (Guo et al., 2011). Constitutive activation of the GTPase KRAS promotes mitogen-activated protein kinase (MAPK) signaling as well as increased dependence on autophagy (Guo et al., 2011). Some types of Ras-driven cancers, such as those with *H-Ras^*V*12^* or *K-Ras^*V*12^* mutations, display up-regulated levels of basal autophagy despite active mTORC1 (Grotemeier et al., 2010; Guo et al., 2011). Signaling through the Ras/Extracellular Signal-Regulated Kinase (ERK) pathway also induces autophagy through BECN1 (Grant, 2008; Mendoza et al., 2011; Wong et al., 2015; Butler et al., 2017). These cancers are considered “autophagy addicted,” as they not only require autophagy in the absence of nutrients but also depend on autophagy for tumor growth (Guo et al., 2011). In these tumors, mTOR can be bypassed as a regulator of autophagy (Perera et al., 2015; Wong et al., 2015).

An illustrative example of *Ras*-driven autophagy addiction is PDAC, a highly aggressive cancer with a near 100% KRAS mutation frequency and a 5-year survival rate of less than 5 percent (Siegel et al., 2018; Waters and Der, 2018). To investigate the interplay between autophagy and *Ras*-mediated tumorigenesis, Guo et al. (2011) transduced non-tumorigenic immortal baby mouse kidney cells (iBMK) with *H-ras^*V*12^* or *K-ras^*V*12^* and evaluated tumor growth in the presence or absence of the key autophagy genes *Atg5* and *Atg7*. The chronic impairment of autophagy significantly reduced tumor formation in nude mice (Guo et al., 2011). Interestingly, in *KRAS^*G*12*D*^*-driven humanized mouse models of pancreatic ductal adenocarcinoma (PDAC), deletion of *Atg5* or *Atg7* leads to development of premalignant pancreatic lesions, while preventing further progression to malignancy (Rosenfeldt et al., 2013; Yang et al., 2014).

In HER2-positive breast cancer, the precise role of autophagy in tumorigenesis and tumor progression is currently being investigated. Recent work has demonstrated that HER2-positive breast cancer cells utilize lower levels of basal autophagy compared to HER2-negative breast cancers under normal conditions, but under stressed conditions, induce autophagy to a greater extent (Bortnik et al., 2016). This differential induction of autophagy was mediated in part through activation of ATG4B, a protease that cleaves pro-LC3B to form LC3-I during autophagosome formation (Bortnik et al., 2016). Interestingly, a recent study by Vega-Rubin-de-Celis et al. (2018) demonstrated a novel mechanism of autophagy suppression via interaction of HER2 with BECN1. HER2-positive breast cancer patients with allelic loss of *BECN1* have worse clinical prognosis, suggesting that suppression of autophagy through this interaction may have pro-tumorigenic effects. Indeed, disruption of this interaction using a small molecule, Tat-Beclin 1, in mice bearing BT-474-VH2 xenografts resulted in increased autophagy induction and reduced tumor progression as effectively as treatment with the tyrosine kinase inhibitor lapatinib (Vega-Rubin-de-Celis et al., 2018).

Autophagy plays a role in nearly every phase of the metastatic cascade, including modulation of tumor cell motility and invasion, cancer stem cell viability and differentiation, resistance to anoikis, epithelial-to-mesenchymal transition, tumor cell dormancy and escape from immune surveillance, and establishment of the pre-metastatic niche (for a recent review on this topic the reader is referred to Mowers et al., 2017). Importantly, autophagy is also upregulated in response to stressful stimuli such as DNA damage induced by cytotoxic agents, contributing to treatment resistance (Kroemer et al., 2010). For example, increased autophagy induction in response to treatment with the HER2-directed therapies trastuzumab and lapatinib has been implicated as a mechanism of drug resistance. Compared to trastuzumab-sensitive SKBR3 breast cancer cells, trastuzumab-resistant JIMT-1 cells constitutively utilize higher levels of autophagy in order to sustain proliferative activities (Cufi et al., 2013). Similarly, treatment of HER2-positive cells with lapatinib has been shown to increase autophagy induction, which, if sustained, allows cells to survive and develop drug-resistance (Tang et al., 2012; Lozy et al., 2014; Chen et al., 2016).

## Promotion of Autophagy Through Nutrient or Energy Restriction

Collectively, the studies described above highlight the context-dependent role of autophagy in cancer incidence and progression. Thus, it may be unsurprising that both autophagy induction and autophagy inhibition have shown promise as viable therapeutic strategies for improving cancer outcomes. Evidence for autophagy induction achieved through nutrient or energy restriction is described below.

### Approaches for and Cellular Impact of Dietary Energy Restriction

Approaches to restricting dietary energy intake include caloric restriction (CR) and fasting. CR is a dietary manipulation which decreases typical (*ad libitum*) caloric intake by 20–40% without incurring malnutrition (Mitchell et al., 2015) and has potent anticancer effects in both developing and established cancer (O’Flanagan et al., 2017). On the other hand, fasting involves short term reduction of caloric intake to 0–500 calories for defined intervals of time, typically while consuming water alone, or, in the case of partial fasting regimens, consuming vegetable broths and/or fruit juices (Wilhelmi, de Toledo et al., 2013). Intermittent fasting regimens involve cycles of short-term reduction of caloric intake in intervals ranging from 1 to 3 days per week. This method encompasses protocols for whole-day fasting, time-restricted feeding, and alternate-day fasting (Tinsley and La Bounty, 2015). Whole day fasting indicates total deprivation from caloric intake for periods typically ranging from 24 to 48 h per week, either consecutively or non-consecutively, with *ad libitum* feeding on remaining days (Tinsley and La Bounty, 2015). Time-restricted feeding regimens define consecutive periods of *ad libitum* feeding that range from 3 to 12 h per day with complete fasting during the remaining hours. Intermittent fasting can also be achieved through alternate day fasting or by following the 5:2 diet. In clinical and preclinical protocols for the 5:2 diet, caloric consumption is restricted to approximately 25% of energetic needs on fasting days, with *ad libitum* feeding on the remaining days of the feeding cycle (Patterson et al., 2015).

**FIGURE 2:**
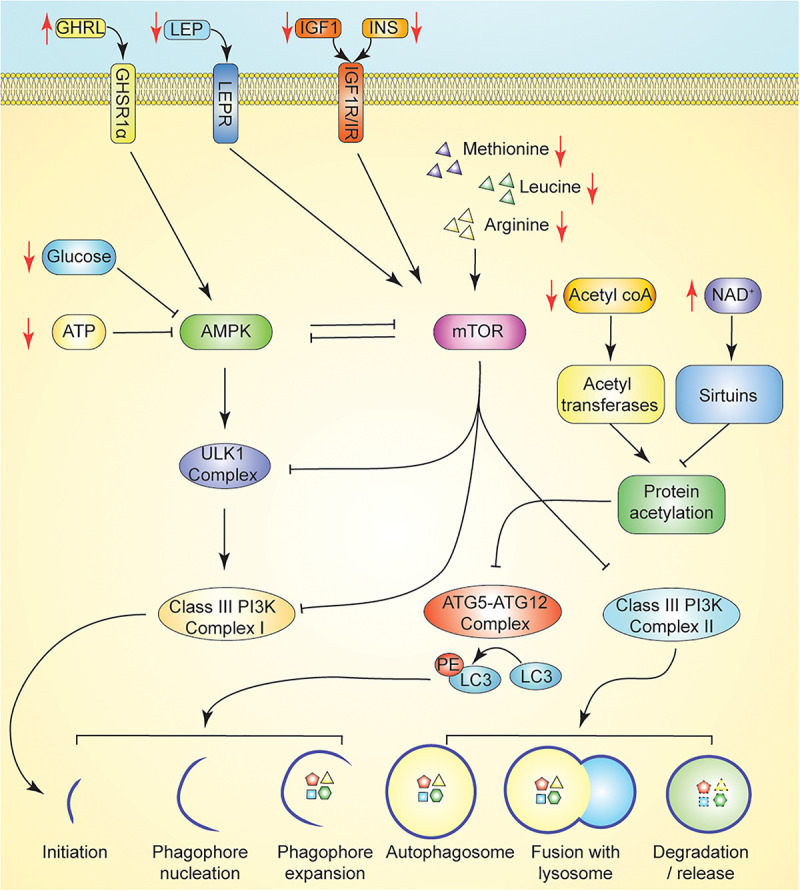
Promotion of autophagy through nutrient or energy restriction. Caloric restriction (CR) suppresses insulin, IGF1, and leptin, each of which suppress autophagy via activation of mTOR following binding to their cognate receptor. Induction of ghrelin by CR promotes activation of AMPK to promote autophagy. Dietary energy modulation by CR limits availability of key nutrient regulators of autophagy including amino acids and glucose. In CR, AMPK signaling is induced in response to reduced ATP and/or glucose concentrations. ATG5-ATG12 complex activity is regulated by the availability of acetyl-coA and the activity of deacetylases. In CR, reduced acetyl-coA limits protein acetylation by reducing substrate availability for acetyltransferases. CR induced increase in NAD + levels promote the activity of sirtuins. Key: Black lines reflect regulation at basal states; Black arrows reflect activation, while black T bars reflect inhibition. Red arrows reflect changes in hormone/metabolite availability induced by fasting which may modulate autophagy.

Under conditions of low nutrient availability, such as those that occur during fasting, autophagic flux is increased in normal and malignant cells to liberate metabolic substrates via degradation of intracellular structures such as damaged proteins and mitochondria. For example, during fasting periods of 12–24 h, mice experience an induction of autophagy in several tissues, including the liver, kidney, and neurons (Komatsu et al., 2005; Alirezaei et al., 2010; Takagi et al., 2016). Specifically, fasting potently activates AMPK in multiple tissues, including skeletal muscle, adipocytes, and the hypothalamus (Figure 2; Kajita et al., 2008; Lim et al., 2010; Bujak et al., 2015). Interestingly, endocrine signaling involving ghrelin, a gut-brain peptide upregulated during periods of fasting, has tissue-specific effects on AMPK activation, as ghrelin activates AMPK in neurons and the hypothalamus yet inhibits AMPK in cardiomyocytes (Figure 2; Toshinai et al., 2001; Andersson et al., 2004; Wang et al., 2014; Bayliss et al., 2016).

Both the ATG12- ATG5- ATG16L and LC3 conjugation systems are regulated by protein acetylation status—further tying autophagic flux to cellular energy and nutrient status (Bánréti et al., 2013). High levels of acetyl CoA, characteristic of a fed, high-energy state, repress autophagy through acetylation of ATG5, ATG7, ATG12, and LC3 by the p300 acetyltransferase (Lee and Finkel, 2009; Mariño et al., 2014). Conversely, increased expression and activity of the NAD^+^-dependent sirtuin 1 (sirt1), inducible by caloric restriction, stimulates autophagy via direct deacetylation of the Atg and LC3 machinery (Figure 2; Cohen et al., 2004; Lee et al., 2008). Fasting and caloric restriction result in an increase in the cellular NAD^+^/NADH ratio, resulting in high concentrations of the NAD^+^ substrate necessary for sirtuin activity (Hayashida et al., 2010). Other sirtuins regulate autophagy indirectly. Sirt2 has been implicated in autophagy modulation through its role in inactivating cytosolic FoxO1, which, under starvation conditions, disassociates from Sirt2 and promotes autophagy via acetylation of lysine residues on Atg7 (Zhao et al., 2010). Sirt3, the primary mitochondrial histone deacetylase, plays a key role in oxidative stress homeostasis through its role in deacetylation of superoxide dismutase 2 (SOD2), a major mitochondrial antioxidant enzyme. Caloric restriction and oxidative stress increase the expression of Sirt3, which is now recognized as a critical component of multiple autophagy inducing pathways (Qiu et al., 2010; Pi et al., 2015; Shi et al., 2015; Liu et al., 2018).

### Systemic Impact of Dietary Energy Restriction

CR and fasting promote longevity in model organisms via reprogramming of endocrine signaling and systemic metabolism, reducing exposure to oxidative stress, and improved mitochondrial function (Michalsen and Li, 2013). Autophagy has been implicated in CR-mediated effects on longevity, and animal models have also demonstrated that this induction of autophagy is necessary for survival during fasting, as it is required to prevent fatal hypoglycemia and cachexia (Karsli-Uzunbas et al., 2014). In the context of cancer, chronic CR has demonstrated tumor suppressive effects in breast, colon, and pancreatic cancers in animal models (Brandhorst et al., 2015; Di Biase et al., 2016; Rossi et al., 2017).

In rodents, fasting and CR modulate similar metabolic targets, but elicit distinct physiological responses (Lee and Longo, 2011). During periods of fasting, serum glucose levels decrease and hepatic glycogen stores diminish within 24 h (Longo and Mattson, 2014). Alternative metabolic pathways are upregulated to provide substrates for energy utilization; for example, gluconeogenesis is activated to provide glucose to specific tissues, primarily the brain. Additionally, β-oxidation of free fatty acids released from adipose tissue is upregulated, while the ketone bodies β-hydroxybutyrate and acetoacetate, released as a byproduct of β-oxidation and from the conversion of ketogenic amino acids, are utilized in the process of ketolysis (Longo and Mattson, 2014). Fasting also results in pronounced endocrine changes, as discussed below. In mice, intermittent fasting regimens are modeled by completely removing food for approximately 24–48 h every 5–7 days (Longo and Mattson, 2014). This intervention decreases fasting insulin, glucose concentrations, total plasma cholesterol, and triglycerides as effectively as continuous CR (Varady et al., 2007). Within a 48 h fasting period, blood glucose decreases by roughly 50% (Jensen et al., 2013).

Metabolic benefits from CR and/or fasting have also been demonstrated in humans. Adherence to these dietary restriction protocols promotes modest weight loss and reductions in total plasma cholesterol and triglyceride concentrations, glucose, and low-density lipoprotein cholesterol (Johnson et al., 2007; Klempel et al., 2013; Rothschild et al., 2014). For example, a 48 h fast in rodents results in weight loss of approximately 20 percent of total body weight, compared to a 4 day fast in humans which results in less than 2 percent weight loss (Pietrocola et al., 2017b). While blood glucose levels in humans decrease after 2 days in the fasted state, clinically acceptable glucose levels are maintained within this period (Lieberman et al., 2008). Additionally, in humans and mice, IGF1 levels decrease by approximately 30 and 70 percent, respectively, during periods of fasting ranging from 24 to 72 h (Dorff et al., 2016). Alternatively, IGF1 decreases by 25 percent with continuous CR in mice, but in humans does not decline unless CR is also accompanied by restriction of protein intake (Lee and Longo, 2011). An intermittent fasting regimen that restricted calories by 85% on alternate fasting days in mice resulted in decreases in IGF1, leptin, and visceral fat, and increased levels of adiponectin (Varady et al., 2009). Thus, a variety of dietary energy restriction approaches are available to reduce circulating IGF1.

A reduction in circulating IGF1 and insulin levels in humans may result in increased autophagic flux through downregulation of the PI3K/AKT/mTOR pathway (Figure 2; Thissen et al., 1994). Leptin, an adipokine, is another known regulator of energy expenditure and neuroendocrine signaling, and is associated with cancer progression (Garofalo and Surmacz, 2006). Leptin has tissue-specific effects on autophagy; however, it is predominantly associated with autophagy inhibition via the PI3K/Akt/mTOR signaling pathway (Maya-Monteiro and Bozza, 2008; Wang et al., 2012; Cassano et al., 2014). In both obese and normal weight humans, fasting and CR also decrease serum concentrations of leptin, consistent with its classical role as a satiety hormone (Boden et al., 1996; Weigle et al., 1997; Rogozina et al., 2011).

Intermittent fasting regimens have not consistently been demonstrated to improve insulin and glucose control (Patterson et al., 2015). One study that compared the metabolic impact of intermittent CR (2 days per week) vs. continuous CR (7 days per week) in overweight, premenopausal women demonstrated that intermittent CR resulted in a greater reduction in fasting insulin levels and insulin resistance (Harvie et al., 2011). In both interventions, similar decreases in leptin, C-reactive protein, LDL cholesterol, and triglycerides were achieved, but the differences in glycemic control that followed adherence to each respective regimen indicate that different mechanisms may be driving the metabolic alterations (Harvie et al., 2011).

### Dietary Energy Restriction During Cancer Therapy

In response to fasting and fasting-mimicking diets, normal cells enter a state characterized by decreased cellular division, reduced metabolic activity, and increased utilization of repair pathways, resulting in chemo-protective effects (Raffaghello et al., 2008; Lee et al., 2010). Decreased levels of bioavailable serum IGF1 and reduced activation of the PI3K/Akt/mTOR axis are implicated in both the longevity effects of CR as well as this fasting-induced stress resistance in normal cells (Raffaghello et al., 2008; Lee et al., 2010). Conversely, as malignant cells are unable to downregulate their oncogene-driven metabolic programs, their sensitivity to chemotherapeutics is retained or even increased following bouts of short-term fasting, resulting in destruction of cancer cells by chemotherapy in a phenomenon termed *differential stress-sensitization* (Raffaghello et al., 2008; Lee et al., 2010).

Though the impact of dietary energy restriction on cancer progression in humans has not yet been fully characterized, interventions which reduce caloric intake during cytotoxic chemotherapy may improve therapeutic efficacy while reducing undesirable side effects in untransformed cells (Buono and Longo, 2018). In humans, side effects from cytotoxic chemotherapies include nausea, vomiting, gastrointestinal inflammation, central and peripheral neurotoxicity and neuropathy, bone marrow toxicities such as myelosuppression and febrile neutropenia, and long-term sequelae including cardiovascular disease and increased risk of secondary malignancies (Nurgali et al., 2018). These side effects are non-trivial and may result in physical and emotional stress that poses an obstacle to treatment, negatively influencing patient outcomes.

Numerous short-term fasting protocols, including intermittent fasting, periodic fasting, and fasting-mimicking diets, have been tested for their ability to improve efficacy and tolerability of chemotherapy cycles (Brandhorst and Longo, 2016). Unlike intermittent fasting, periodic fasting regimens last for 3 days or longer and are repeated every 2 or more weeks, while fasting-mimicking regimens use a plant-based low carbohydrate and low-protein diet that is indicated for use every 3 to 4 weeks (Longo and Mattson, 2014; Brandhorst et al., 2015). There are numerous clinical trials registered on ClinicalTrials.gov investigating the impact of fasting or other dietary energy restriction approaches on response to chemotherapy across a wide variety of cancer types. Most of these trials to date have focused on tolerability of the fasting or fasting-mimicking regimen in combination with chemotherapy as well as measurable side effects in human subjects. We will discuss below representative trials for which final or interim results have been peer-reviewed for publication or submitted as abstracts for presentation at major conferences.

Bauersfeld et al. (2018) conducted a randomized, individually controlled cross-over trial wherein subjects with breast and ovarian cancers underwent a modified fasting protocol for multiple 60 h periods over the course of three out of six cycles of chemotherapy (36 h before to 24 h after the chemotherapy; subjects were fasted during either the first three cycles or the second three cycles). Subjects were allowed a maximum daily intake of intake of 350 kcal during fasting periods and reported improved quality-of-life and reduced self-reported fatigue following therapy when therapy was administered during a fasting period (Bauersfeld et al., 2018). Greater benefit was perceived when subjects were fasted during the first three chemotherapy cycles as opposed to the second three cycles (Bauersfeld et al., 2018).

Cheng et al. (2014) reported a protective effect of prolonged fasting cycles during chemotherapy against chemotherapy-induced myelosuppression in mice, as well as preliminary findings suggesting myeloprotective effects of fasting in humans. Similarly, de Groot et al. (2015) investigated whether fasting for 24 h before receiving (neo) adjuvant TAC-chemotherapy therapy and for a subsequent 24 h after completing therapy could reduce hematological toxicity in subjects with stage II and III HER2-negative BC, using γ-H2AX in peripheral blood mononuclear cells (PBMCs) as a proxy marker for chemotherapy toxicity in normal somatic cells. No significant differences were observed in the frequency of grade I, II, III, or IV side effects due to fasting; however, fasted subjects experienced attenuated bone marrow toxicity as well as a smaller and less consistent increase in markers of chemotherapy-induced DNA damage in PBMCs compared to non-fasted subjects (de Groot et al., 2015). Of note, while fasting significantly reduced IGF1 as compared with baseline values, final IGF1 serum values did not differ across the two treatment arms (de Groot et al., 2015). Similar findings suggesting protection against bone marrow toxicity and DNA damage in circulating PBMCs were also reported following prolonged fasting (48–72 h) in subjects receiving platinum-based combination chemotherapy without concurrent radiation across a variety of cancer types (Dorff et al., 2016). Limitations of this study include a small sample size and the lack of a non-fasted control group (Dorff et al., 2016). Importantly, the safety of completely abstaining from food for periods of 2 or more days has been demonstrated in a medically-supervised setting with the majority of cancer patients experiencing minimal adverse reactions (de Groot et al., 2015; Bauersfeld et al., 2018; Finnell et al., 2018).

Taken together, the quantitative biomarker-based data available to support fasting-induced differential stress resistance in humans during chemotherapy is limited but compelling. Considering the importance of autophagy in protection against genotoxic insult and cellular transformation, future studies should address whether autophagy induction in normal cells underlies the reduced severity of chemotherapy-induced side effects and/or increased rate of cellular repair in normally functioning cells in response to dietary restriction.

### Dietary Energy Restriction in Cancer Cachexia

Cancer cachexia is a catabolic wasting syndrome characterized by anorexia and progressive loss of muscle and adipose tissue mass (Aoyagi et al., 2015). The combination of hypermetabolism and the anorectic effect of elevated IL-6 contribute to a chronic caloric deficit of approximately 200-450 kcals per day in weight-losing patients with cachexia (Kumar et al., 2010; White, 2017). Elevated IL-6 also leads to the release of glucocorticoids, which contribute to muscle wasting in cancer cachexia (White, 2017). While parenteral nutritional supplementation provides some benefit, as does enteral tube feeding (Amano et al., 2020), there is little effect on mortality in response to oral dietary supplementation (Baldwin et al., 2012).

Given the strong relationship between caloric intake and mortality in patients with cachexia, CR/fasting interventions in patients with advanced cancers may not be advised. Some of the metaboendocrine effects of CR—including improved insulin sensitivity, reduced leptin, and increased ghrelin—have been independently considered as approaches for intervention in cancer cachexia. For example, treatment with ghrelin has arisen as a promising treatment option in cancer cachexia, improving appetite, food consumption, and body composition (Khatib et al., 2018). While CR is associated with elevation of ghrelin, by definition of CR this elevation cannot translate into improved caloric intake. Additionally, low leptin predicts poor survival in cancer cachexia (Mondello et al., 2014), while induction of autophagy in response to CR contributes to muscle wasting in mouse models of cachexia (Penna et al., 2013, 2019).

Other approaches used to combat cachexia have included several immunomodulatory agents, which dampen proinflammatory signaling (Aoyagi et al., 2015). While inflammatory signaling pathways are reduced by CR, growth factor signaling is also reduced (Hursting et al., 2013); thus it is unclear whether the anti-inflammatory aspects of CR promote or impair retention of skeletal muscle mass, or perhaps even further exacerbate wasting. In an experimental model of cachexia, CR preserved grip strength but did not otherwise alter the course of cachexia (Levolger et al., 2018). It should also be noted that in this study CR was not compared against other protective interventions (Levolger et al., 2018). In summary, while CR may appear to promote a protective metaboendocrine state, limited evidence support a protective role for CR and much of the existing literature implicate CR as a potentially deleterious intervention in the context of cachexia. Thus, any consideration of CR or fasting in cancer therapy should include assessment of the patient’s risk of cachexia.

## Pharmacological Autophagy Modulation as an Approach to Cancer Treatment

### Perturbing Growth Factor Signaling as a Mimetic of Dietary Energy Restriction

**FIGURE 3:**
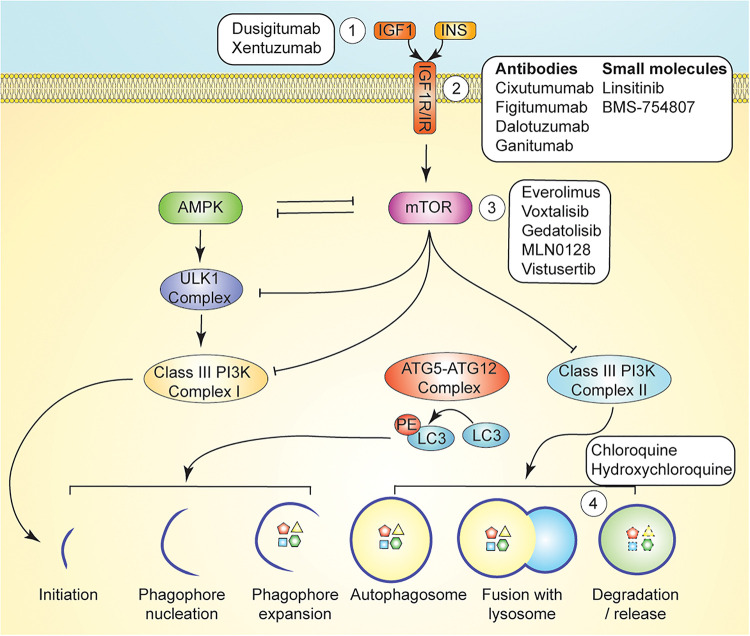
Pharmacological Autophagy Modulation. mTOR signaling regulates induction of autophagy via inhibition of AMPK, ULK1, and Class III PI3K signaling. Autophagy can be induced by inhibition of growth factor signaling upstream of mTOR by ligand-targeting monoclonal antibodies (Kuma and Mizushima, 2010), receptor-targeting monoclonal antibodies, or small molecule inhibitors (e.g., AXL1717 and BMS-754807) (Mizushima et al., 2004). mTOR signaling is directly inhibited with compounds such as temsirolimus, and everolimus (Shaid et al., 2013). Autophagy can be inhibited with lysosomotropic agents such as chloroquine and hydroxychloroquine, which inhibit maturation of the autophagolysosome (Mizushima and Komatsu, 2011).

Reductions in circulating IGF1 may be an important driver of the potent anticancer effects of dietary restriction, as fasting and CR result in enhanced cancer cell apoptosis, reduced angiogenesis, and alterations in key metabolites and systemic signaling pathways downstream of IGF1/IGF1R (O’Flanagan et al., 2017). As a reduction in bioavailable IGF1 is a common theme in response to dietary energy restriction interventions, it is tempting to speculate that inhibitors of IGF1 signaling could be used as a metabolic reprogramming intervention and mimetic of energy restriction (Figure 3), yielding some of the protective effects of fasting on chemotherapy toxicity. IGF1 is a nutrient-sensitive endocrine hormone that is primarily secreted by the liver. Upon binding of IGF1 to its cognate receptor, insulin-like growth factor receptor 1 (IGF1R), autophosphorylation events lead to the activation of two signaling axes—MAPK and PI3K (class I)/AKT/mTOR—which promote increased cell proliferation, inhibition of autophagy, and evasion of cell death (Meynet and Ricci, 2014; O’Flanagan et al., 2017). Human studies have demonstrated that modest protein restriction in a chronic CR regimen modulates anti-cancer effects associated with decreased IGF1 levels (Fontana et al., 2008). However, monoclonal antibodies directed at IGF1R have resulted in unexpected toxicity in human subjects when combined with chemotherapy (Langer et al., 2014; Di Cosimo et al., 2015; Baselga et al., 2017), while several small molecule inhibitors of IGF1R have not yielded clinical benefit when used as single agents in clinical trials (Fassnacht et al., 2015; Chiappori et al., 2016; Gradishar et al., 2016; Bergqvist et al., 2017). Yet, small molecule IGF1R inhibitors—as well as inhibitors of other components of the IGF1R pathway—may still hold clinical potential when used in combination therapies. For example, combination of AXL1717 (picropodophyllin), an IGF1R pathway inhibitor, with gemcitabine HCl and carboplatin yielded an acceptable toxicity profile in previously untreated, locally advanced, or metastatic NSCLC (Holgersson et al., 2015). Similarly, BMS-754807 is a reversible small molecule inhibitor of IGF1R and insulin receptor (IR) (Carboni et al., 2009) that has demonstrated effectiveness *in vitro* in combination with anti-cancer therapies for the treatments of breast, pancreatic, colon, lung, and gastric cancers (Carboni et al., 2009). IGF1R inhibition may also be an approach to tackling drug resistance in HER2-overexpressing breast cancers, as one of the potential mechanisms of resistance to trastuzumab occurs through upregulation of IGF1R and subsequent cross-phosphorylation and activation of HER2 (Chakraborty et al., 2017).

Direct inhibition of mTOR has also been investigated as an approach to manipulation of the PI3K/Akt/mTOR axis (Figure 3). Combination of the PI3K/mTOR inhibitor buparlisib with fulvestrant resulted in a significant increase in median progression-free survival yet an unacceptable toxicity profile in postmenopausal women with hormone-receptor-positive, HER2-negative, advanced breast cancer (Di Leo et al., 2018). However, mTOR inhibition via temsirolimus or everolimus, a derivative of rapamycin that inhibits activation of mTORC1 by binding to FKBP12 (also known as RAD001 or Affinitor), in combination with liposomal doxorubicin and bevacizumab was well tolerated and yielded an increase in objective response rate in patients with metaplastic TNBCs bearing PI3K/AKT/mTOR pathway aberrations (Basho et al., 2017).

Treatment with everolimus downregulates the nutrient-sensing effects of mTOR and results in reduced protein synthesis, cellular proliferation, and glucose uptake, as well as increased autophagic flux (Jobard et al., 2017). In HER2-positive breast cancer patients treated with trastuzumab plus everolimus, serum metabolomic analysis revealed that this combination modulated a physiological state similar to that which occurs during fasting where lipolysis and autophagy are upregulated and gluconeogenesis and glycogenolysis are decreased (Jobard et al., 2017). The combination of everolimus with HER2-directed therapies is also a promising approach to combat drug resistance. In drug resistant HER2-positive breast tumors, trastuzumab treatment increases phosphorylation of PDK1 and mTOR, which activates ribosomal protein S6 kinase beta-1 (S6K1) and promotes anabolic activities (Huynh et al., 2017). Blocking this escape pathway with everolimus is one approach to improve the efficacy of trastuzumab.

### Metabolic Reprogramming Interventions (MRIs)

In addition to direct inhibition of growth factor signaling, a pharmacological strategy currently being investigated to treat cancer involves the combination of metabolic reprogramming interventions (MRIs) with traditional cytotoxic chemotherapies. These approaches are based on the identification of compounds that mimic the beneficial effects of caloric restriction without the need for challenging dietary changes. A subclass of MRIs is termed caloric restriction mimetics (CRMs), which induce a metabolic reprogramming in cancer cells intended to recapitulate the biochemical effects of dietary energy restriction.

CRMs exert their anticancer effects by increasing autophagic flux in response to a reduction in cellular protein acetylation (Madeo et al., 2014). This increase in autophagic flux results in an increase in extracellular ATP, a potent chemoattractant for professional phagocytes (Corriden and Insel, 2012), and therefore promotes immunogenic cell death. Pietrocola et al. (2016) provide compelling evidence that robust immunosurveillance and increased chemotherapeutic efficacy following nutrient deprivation are dependent upon an increase in cancer cell autophagy; the increased chemosensitivity and enhanced immunity were reversible upon intraperitoneal injection of recombinant IGF1). Importantly, success of antineoplastic therapies in the long-term is largely determined by their ability to reinstate robust and prolonged anticancer immunosurveillance (Kepp et al., 2014).

### Pharmacological Inhibition of Autophagy

Despite gaps in our understanding of autophagy’s complete role in cancer, pharmacological inhibition of autophagy is currently being investigated for potential use as adjuvant therapy, as inhibition of autophagy causes metabolic instability that can be exacerbated in combination with therapy (Pascolo, 2016). Chloroquine (CQ) is a pharmacological agent that indirectly inhibits autophagy by preventing endosomal acidification, resulting in inhibition of lysosomal enzymes that require an acidic pH and disrupting the maturation of the autophagolysosome (Figure 3; Solomon and Lee, 2009; Amaravadi et al., 2011). In preclinical models of Ras-driven pancreatic cancers, CQ has been shown to effectively reduce cell growth, tumorigenicity, and oxidative phosphorylation (Yang et al., 2011). Yang et al. (2011) demonstrated that CQ potently retards *in vitro* proliferation and anchorage-independent growth of several different human pancreatic cell lines. Moreover, CQ treatment significantly increased survival in a transgenic, Kras-driven murine model of PDAC and diminished *in vivo* growth of a human PDAC cell line in immunocompromised mice (Yang et al., 2011).

However promising, the translational relevance of these findings is limited. Subcutaneous injection of pancreatic cell lines precludes investigation into factors within the pancreatic tumor microenvironment that may hinder or promote tumor cell survival in the face of autophagy ablation. Similarly, the use of immunocompromised mice prevents identification of potentially important effects of autophagy inhibition on tumor immunosurveillance (Li Y. Y. et al., 2017; Pietrocola et al., 2017a). Of note, a phase II pharmacodynamic study that used the chloroquine analog hydroxychloroquine (HCQ, which has been shown to have decreased toxicity in humans compared to CQ) to inhibit autophagy in patients with metastatic PDAC showed no significance in progression-free survival (Wolpin et al., 2014).

Many chemotherapies—such as gemcitabine, which is commonly used to treat PDAC, or platinum-based compounds used in the treatment of primary and metastatic breast cancers—induce autophagic flux, and the putative cytoprotective roles of autophagy may limit the efficacy of chemotherapy (Donohue et al., 2013; Hashimoto et al., 2014; Jiang et al., 2017). Therefore, CQ in combination with chemotherapy may present an attractive therapeutic strategy to increase the cytotoxicity of treatment regimens (Hashimoto et al., 2014). Indeed, combination treatment with chloroquine and gemcitabine showed increased efficacy in delaying tumor growth of patient-derived PDAC xenografts relative to the use of either as a single agent (Balic et al., 2014). A phase I clinical trial in patients with metastatic or unresectable PDAC reported no dose-limiting toxicities following a combination of CQ and gemcitabine; furthermore, of the nine patients enrolled in the trial, 3 patients showed a partial response while two patients exhibited stable disease (Samaras et al., 2017). At the time of this review, the Abramson Cancer Center of the University of Pennsylvania is actively recruiting patients with advanced primary or metastatic PDAC to explore the efficacy of hydroxychloroquine in combination with gemcitabine or another chemotherapeutic agent (ClinicalTrials.gov: NCT01506973). Collectively these findings suggest that CQ and its analogs may have the potential to improve clinical outcomes in PDAC treatment when used in combination with current standard-of-care chemotherapy approaches.

Consistent with findings in PDAC, CQ-associated increases in therapeutic potency of chemotherapeutic agents have also been reported in preclinical TNBC studies. Gemcitabine induced mTOR-independent autophagy in MDA-MB-231 cells *in vitro*; accordingly, combination treatment with CQ and gemcitabine resulted in increased apoptotic cell counts compared to treatment with only gemcitabine (Chen et al., 2014). Similarly, a model of human TNBC using subcutaneous patient derived xenografts (PDX) in nude mice reported that the addition of CQ potentiated the effects of adriamycin and cyclophosphamide treatment by significantly reducing primary tumor size and multiplicity of lung metastases (Lefort et al., 2015).

Chloroquine and its analogs have also shown promise in situations of acquired therapeutic resistance, a frequent challenge faced in TNBC treatment (Kim et al., 2018). Compared with the parental MDA-MB-231 cell line, anthracycline-resistant MDA-MB-231 cells showed heightened levels of basal autophagy (Chittaranjan et al., 2014), prompting Chittaranjan et al. (2014) to test whether the use of autophagy inhibition could improve outcomes in cases of therapy resistance. Indeed, combination treatment with epirubicin and HCQ increased therapeutic efficacy by significantly reducing PDX tumor growth compared with saline controls and epirubicin alone (Chittaranjan et al., 2014). CQ in combination with carboplatin also reduced tumor growth in carboplatin-resistant TNBC orthotopic xenografts, potentially through depletion of cancer stem cells (Liang et al., 2016).

### Additional Considerations of Autophagy Modulation

Several autophagy inhibitors are available, and their mechanisms, and potential for modulation of pathways other than autophagy, are distinct. In addition to its ability to prevent completion of the autophagic process, CQ has been implicated in tumor vessel normalization (Maes et al., 2014), suppression of macrophage endocytosis to improve nanoparticle delivery (Wolfram et al., 2017), and increased sensitivity to cisplatin in breast cancer cells (Maycotte et al., 2012), each of which were shown to occur via mechanisms independent of autophagy suppression. With this in mind, based on promising preclinical data, there are a growing number of clinical trials investigating the potential for use of CQ as adjuvant therapy, including a Phase II trial testing the efficacy and safety of CQ in combination with taxane or taxane-like chemotherapeutic agents in the treatment of advanced or metastatic BC who were non-responders to AC therapy (ClinicalTrials.gov; NCT01446016).

Perhaps in the context of chemotherapeutic resistance, an approach combining cancer therapies with interventions that increase dependence on autophagy (e.g., through manipulation of dietary energy intake or pharmacologic interventions such as MRIs or growth factor inhibition) will expose a metabolic weakness that could be exploited with autophagy inhibitors. Indeed, the results of Lashinger et al. (2016) showed that conditions of CR in combination with genetic autophagy ablation in a *Ras*-driven model of pancreatic cancer had greater effects on decreasing tumor volume and progression than either condition in isolation. The effect of chemotherapy under these conditions was not explored. Combining autophagy induction and inhibition increased radiosensitivity of colorectal cancer cells in culture (Shiratori et al., 2019). However, a phase 1 trial combining the Akt inhibitor MK-2206 with hydroxychloroquine in patients with advanced solid tumors resulted in a substantial number of drug-related adverse events and minimal evidence of antitumor activity (Mehnert et al., 2019). Interestingly, use of these drugs in combination altered the pharmacokinetics of both drugs (Mehnert et al., 2019), which may have impacted toxicity. Perhaps in combination the dosages of these drugs should be reduced, or hydroxychloroquine should be tested in combination with other autophagy inducers.

Notably, some have reported that cancer cell autophagy is required for immunogenic cell death yet dispensable for chemotherapy-induced cell death (Michaud et al., 2011). Antunes et al. (2017) demonstrated increased chemotherapeutic efficacy following nutrient deprivation in an autophagy-independent manner. These findings argue for caution regarding the use of autophagy inhibitors in the absence of chemotherapeutic resistance, as an inhibition of primary tumor growth may be concomitant with impairment of anti-tumor immunosurveillance and an elevated risk of recurrence. Longitudinal resection studies in mice addressing the potential for recurrence following autophagy inhibition during treatment may be helpful in untangling this research question.

## Conclusion

In sum, cancer cells often exhibit an increased dependence on autophagy, both for baseline survival and as a response to stressors such as chemotherapy or a decline in nutrient availability. Numerous hormonal and metabolic cues direct autophagic induction in cancer. There is evidence to suggest that the increased dependence on autophagy in cancer cells may be exploitable clinically by combining autophagy modulators with existing chemotherapies. Fasting appears to hold promise for reducing dose-limiting side effects of chemotherapy in humans. However, it remains unclear whether therapeutic agents aimed at autophagy induction, autophagy inhibition, or both are a viable therapeutic strategy for improving cancer outcomes. In light of the burgeoning interest in precision medicine, identification of oncogenic drivers associated with increased susceptibility to fasting, autophagy induction or inhibition may hold clinical promise.

## Author Contributions

AC contributed to concept development, writing, preparation, figure design, and submission of this article. MC contributed to concept development, writing, preparation, and figure design. JP, AP, and SE contributed to concept development and writing. SH contributed to concept development, writing, preparation, figure design, and submission of this article. All authors contributed to the article and approved the submitted version.

## Conflict of Interest

The authors declare that the research was conducted in the absence of any commercial or financial relationships that could be construed as a potential conflict of interest.
